# Reasonable Planting Density and Chemical Regulation Can Improve the Plant Morphological Characteristics of Grain Maize, Enhance Lodging Resistance and Increase Yield in the Hexi Oasis

**DOI:** 10.3390/plants15101558

**Published:** 2026-05-20

**Authors:** Wei Pan, Haoliang Deng, Fuqiang Li, Weijie Shi, Jianlong Wei, Qinli Wang, Xiaofan Pan, Wenbo He

**Affiliations:** 1 Gansu Provincial Engineering Research Center for the Resource Utilization of Edible Fungi and Fungi Bran, Hexi University, Zhangye 734000, China; panwei2025666@163.com (W.P.); wangqinli66@163.com (Q.W.); panxiaofan2023@163.com (X.P.); 2 College of Water Conservancy and Hydropower Engineering, Gansu Agricultural University, Lanzhou 730070, China; lifuq@gsau.edu.cn (F.L.); hewenbo2025@163.com (W.H.); 3 College of Agriculture and Ecological Engineering, Hexi University, Zhangye 734000, China; 4 Agricultural Technology Extension Center of Ganzhou District, Zhangye 734000, China; shiweijie202688@163.com (W.S.); weijianlong2026@163.com (J.W.)

**Keywords:** planting density, chemical regulation measures, lodging resistance, leaf structure, yield, grain maize

## Abstract

To investigate the effects of planting density and chemical regulation measures, as well as their interactions, on the plant morphological characteristics, stem mechanical properties, leaf anatomical structure, dry matter accumulation and allocation, and yield and its components of grain maize in the Hexi Oasis irrigation area, a field experiment was conducted from 2024 to 2025. Planting density was set as the main factor, with five density levels: 82,500 plants·ha^−1^ (M1), 97,500 plants·ha^−1^ (M2), 112,500 plants·ha^−1^ (M3), 127,500 plants·ha^−1^ (M4) and 142,500 plants·ha^−1^ (M5). Chemical regulation measures were set as the secondary factor, consisting of two treatments: spraying 30% aminoethyl hexanoate·ethephon at the 10-leaf stage (T1) and spraying an equal amount of water as the control (T2). The results revealed that, as planting density increases, the maize plant height, ear height and stem breakage rate rise continuously, whilst stem diameter, stem breaking resistance, rind puncture strength, leaf thickness and epidermal tissue thickness showed a downward trend. The leaf area index, ear length, kernel number per ear, kernel weight and yield all exhibited a trend of first increasing and then decreasing, reaching their peak at the M3 planting density. Compared with conventional planting patterns, spraying chemical regulators significantly reduced plant height by 10.66~13.99% and ear height by 16.12~19.57%, increased stem diameter by 2.12~13.79%, and enhanced stem breaking resistance by 7.71~23.11% and rind puncture strength by 5.17~12.65% at 30 days after silking. Additionally, it delayed leaf senescence, increased the leaf area index by 4.37~10.03% during the filling stage, and increased yield by 1.99~4.06%. The synergistic effect of moderately increasing planting density combined with chemical regulation can effectively coordinate the ‘population–individual’ contradiction in maize, reduce plant height and ear height and increase stem diameter and rind puncture strength, while maintaining a higher leaf area index after the silking stage and promoting dry matter translocation to grains, thereby achieving a synergy between lodging resistance and high yield. Among them, a planting density of 112,500 plants·ha^−1^ combined with spraying chemical regulators yielded the highest maize yield and harvest index, reaching 20.28~20.48 t·ha^−1^ and 0.52~0.53, respectively. Compared with other treatments, the increases ranged from 2.54~47.51% for yield and from 1.92~36.84% for the harvest index. Meanwhile, this treatment exhibited superior stem mechanical properties and a lower stem breakage rate. Taking into account factors such as lodging resistance, yield, dry matter accumulation and allocation, it has been determined that a planting density of 112,500 plants·ha^−1^ combined with spraying 30% aminoethyl hexanoate·ethephon at the 10-leaf stage is an effective strategy for achieving both lodging resistance and high yield in grain maize in the Hexi Oasis irrigation area.

## 1. Introduction

Maize (*Zea mays* L.) is a crucial food, feed and energy crop in China, playing a pivotal role in ensuring food security [1]. With population growth and the rapid development of the livestock industry, the demand for maize has been continuously increasing. Therefore, achieving sustained growth in overall maize yield on limited arable land resources is of great significance for realizing increased grain production and efficiency. Increasing planting density is one of the key approaches to unlocking the yield potential of maize [2], and numerous studies have confirmed that increasing the number of ears per unit area can enhance population-level yield [3]. However, planting density exerts multiple regulatory effects on maize growth and development [4]. An appropriate planting density can optimize population structure, enhance light efficiency, promote dry matter accumulation, and thereby achieve high yield [5]. However, when planting density exceeds the threshold, individual plants have less space to grow, and competition for water, nutrients and light between plants intensifies, which leads to poor individual development. This results in etiolated stem elongation, reduced stem diameter and elongated internodes, as well as alterations in plant morphology, physiological characteristics, and canopy structure, thereby affecting light use efficiency and final yield formation [6,7]. Meanwhile, high-density planting significantly increases the risk of stem lodging [8], which not only causes difficulties in mechanical harvesting, but also leads to yield reduction and grain quality deterioration [9,10]. Studies have shown that lodging reduces maize yield by 5~25%, and in some cases by as much as 50% or more [11]. Consequently, it is an urgent priority for high-yield maize planting to optimize plant traits, enhance stem mechanical strength, and achieve lodging resistance under high planting density.

Stem lodging resistance is jointly influenced by plant morphological characteristics, anatomical structure and mechanical properties [12,13]. In terms of morphological characteristics, plant height, ear height and the ear-to-plant height ratio are important indicators for evaluating the stability of the plant’s center of gravity; excessive plant height and ear height cause the center of gravity to shift upwards, making the plant susceptible to lodging after irrigation or rainfall [14]. In terms of anatomical structure, the stem’s mechanical strength is primarily related to the thickness of the sclerenchyma tissue, the number and distribution of vascular bundles, and cortex thickness, which determine the stem puncture strength and breaking resistance [15]. In terms of mechanical properties, the stem breaking resistance and puncture strength are key indicators that directly reflect stem mechanical strength and are significantly negatively correlated with lodging rates [16]. To mitigate the risk of lodging caused by plant density, the application of plant growth regulators has attracted widespread attention [17]. Currently, a commonly used chemical regulator in production is 30% aminoethyl hexanoate·ethephon, which regulates the growth and development process primarily by modulating endogenous hormone levels in plants. Its mechanism of action involves inhibiting gibberellin synthesis, slowing the rate of internode elongation, reducing plant height and ear height, promoting secondary stem growth, increasing stem diameter and wall thickness, and enhancing the deposition of lignin and cellulose in stem tissues, thereby improving mechanical strength [18,19]. Research by Li et al. [20] confirmed that spraying chemical regulators at the appropriate stage reduced maize plant height and ear height by 25.2% and 33.8%, respectively, and significantly increased stem breaking resistance, thereby reducing the lodging rate. However, the application effect of chemical regulators is related to planting density. Under different planting density conditions, the regulatory effects of chemical regulators on plant morphology, stem mechanical properties and overall yield vary [21]. Therefore, coupling planting density and the application rate of chemical regulators is the key approach to achieving the synergy of plant lodging resistance and high yield.

As the primary organ of photosynthesis, the growth and development morphology of leaves directly influences dry matter accumulation and yield formation [22]. The leaf area index is a key indicator of population photosynthetic potential, and an appropriate leaf area index ensures sufficient light and promotes high yield [23]. However, overcrowded planting causes canopy closure, resulting in insufficient light exposure for the middle and lower leaves, which accelerates leaf senescence, leading to a sharp decline in the leaf area index during the late filling stage and shortening the photosynthetic functional period [24]. Meanwhile, leaf anatomical structure is sensitive to planting density, and leaf thickness and epidermal tissue thickness decrease with increasing density, which may affect leaf photosynthetic efficiency and stress tolerance [25]. Studies have shown that chemical regulation measures can optimize plant architecture, improve canopy light distribution, and maintain leaf function [26]. Although optimizing canopy structure and maintaining leaf photosynthetic function can accumulate higher biological yield, whether a higher economic yield can be achieved under high planting density depends on the efficient translocation and distribution of photosynthetic products to the grain [27]. Consequently, yield levels are closely related to the efficiency of dry matter allocation to the grain [28]. Studies have shown that overcrowded planting leads to excessive vegetative growth and relatively insufficient reproductive growth, resulting in a lower harvest index and the phenomenon of ‘large source, small sink’, which limits the realization of yield potential [29]. Therefore, synergistically increasing the population dry matter accumulation and harvest index is the key to achieving high yield under high planting density.

Located in the central part of the Hexi Corridor, Zhangye City has a typical temperate continental arid climate, with abundant light and heat resources, a large diurnal temperature range, and a well-developed irrigated agriculture sector, making it an ideal region for maize production. With the large-scale and mechanized development of maize production, there is a higher requirement for varieties with lodging resistance and population uniformity. Based on this, the present study was conducted in the Hexi Oasis irrigation area, primarily investigating the interactive effects of planting density and chemical regulation measures on the morphological characteristics of maize plants, leaf growth and development, stem mechanical properties, dry matter accumulation and allocation, yield, and yield components. We proposed the following research hypotheses: (1) Increasing planting density promotes stem elongation and reduces stem mechanical strength, thereby increasing lodging risk. However, chemical regulation mitigates these negative effects by inhibiting internode elongation and enhancing stem structural reinforcement. (2) There exists an optimal planting density range within which chemical regulation can simultaneously optimize canopy structure, maintain leaf photosynthetic function, and improve dry matter transport to kernels, thus synergistically enhancing lodging resistance and kernel yield. (3) The positive effects of chemical regulation on plant architecture, stem strength, and harvest index vary with planting density, with greater efficacy under medium-to-high planting densities than under low planting density. This study aims to elucidate the synergistic mechanisms among plant morphological characteristics, stem mechanical properties and yield under the interaction of density and chemical regulation, thereby providing a theoretical basis for high-yield maize cultivation in this region.

## 2. Results and Analysis

### 2.1. Plant Height, Ear Height and Ear-to-Plant Height Ratio

**Figure 1:**
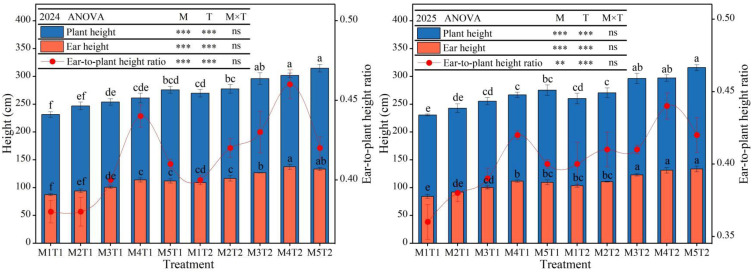
Effects of planting density and chemical regulation on plant height, ear height and ear-to-plant height ratio of grain maize. Bars and error bars stand to represent averaged values ± standard errors (n = 3); different lowercase letters indicate significant differences among the treatments (*p* < 0.05); and ** and *** indicate significant differences among the different treatments at the levels of *p* < 0.01 and *p* < 0.001. ns means not significant at the level of *p* ≥ 0.05.

The plant height, ear height and ear-to-plant height ratio of grain maize were closely related to planting density and the application of chemical regulators, exhibiting similar variation patterns across the experimental years. Furthermore, analysis of variance revealed that planting density and chemical regulation measures had a highly significant effect (*p* < 0.01) on maize plant height, ear height and ear-to-plant height ratio, but there was no significant interaction effect (*p* > 0.05), indicating that planting density and chemical regulation measures exert relatively independent regulatory effects on maize plant growth traits (Figure 1). An analysis of the average data from the two growing seasons revealed that, under the same planting density, the chemical regulation treatments significantly reduced plant height and ear height. Specifically, from M1 to M5, plant height decreased by 10.66~13.99%, and ear height decreased by 16.12~19.57%, while the reduction in the ear-to-plant height ratio was smaller, ranging from 3.57% to 8.43%, with no significant differences. Under both chemical regulation measures and conventional planting conditions, as planting density increases, grain maize plant height exhibited a continuous increasing trend, whilst ear height and the ear-to-plant height ratio exhibited a trend of first increasing and then decreasing. Compared with M1, the increases in maize plant height under M2 were only 5.95% and 3.40%, respectively, with no significant difference, whereas under M3 to M5, the increases ranged from 10.22% to 19.29% and 11.72% to 18.97%, respectively; the increases in ear height ranged from 8.34% to 29.11% and 7.07% to 25.74%, respectively. For the ear-to-plant height ratio, the increases under M2, M3, and M5 were only 2.70~9.46% and 3.75~5.00%, respectively, with no significant difference, whereas under M4, the increases reached 16.22% and 12.50%, respectively. Overall, under different planting densities, chemical regulation measures can effectively regulate the plant height and ear height of grain maize, lowering the plant’s center of gravity, optimizing population structure, and enhancing the stem lodging resistance.

### 2.2. Leaf Area Index

**Figure 2:**
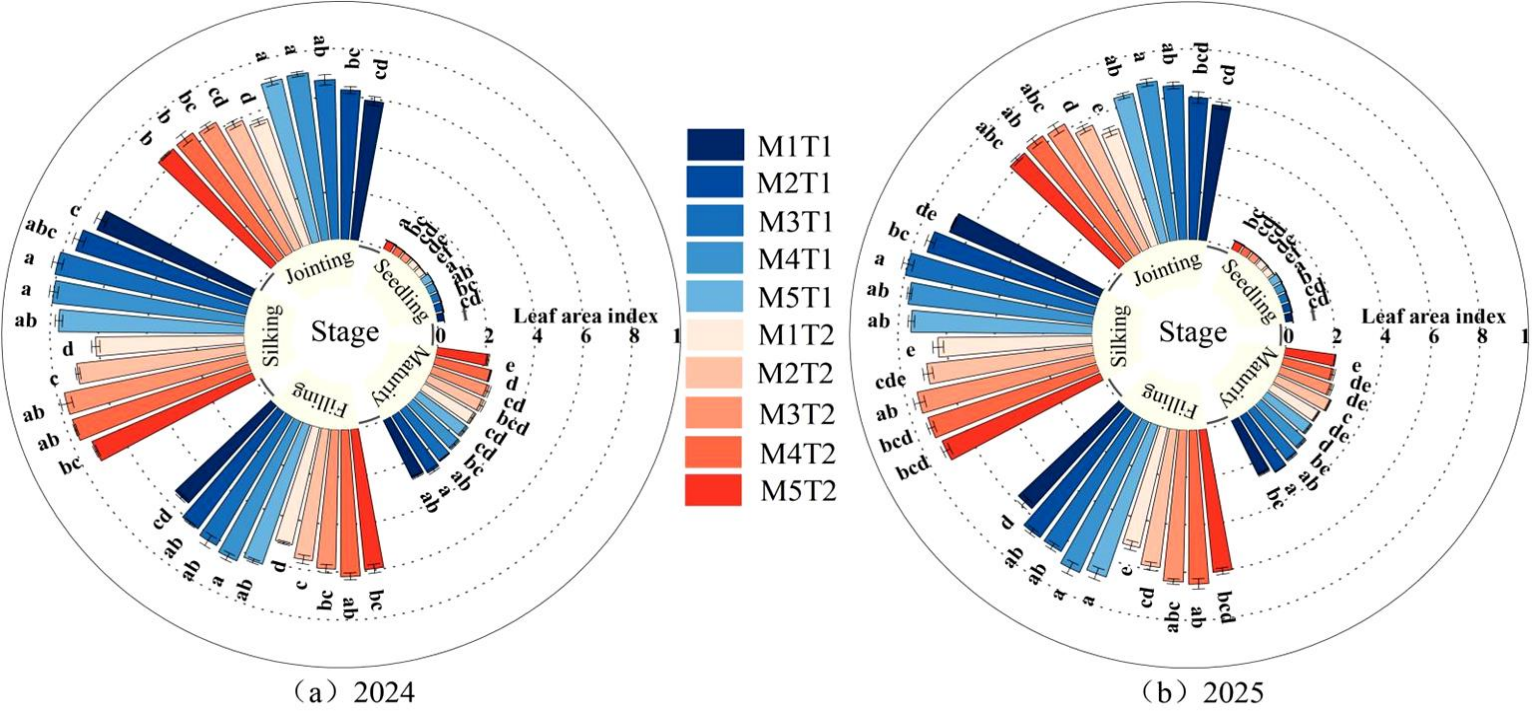
Effect of planting density and chemical regulation on leaf area index of grain maize. Bars and error bars stand to represent averaged values ± standard errors (n = 3); different lowercase letters indicate significant differences among the treatments (*p* < 0.05).

The leaf area index of grain maize exhibited a unimodal curve, peaking at the silking stage, and different density growth–chemical regulation treatments had a significant effect on the leaf area index of grain maize (Figure 2). The results of the variance analysis revealed that both planting density and chemical regulation measures had a highly significant effect (*p* < 0.01) on the leaf area index from the jointing stage to the maturity stage, but the interaction had no significant effect (*p* > 0.05) (Table 1). An analysis of the average data from the two growing seasons revealed that, under the same planting density, chemical regulation measures increased the leaf area index compared with conventional planting conditions, with increases of 2.96~7.09%, 5.26~8.57%, 4.63~10.49%, 4.37~10.03%, and 12.01~12.95% at the seedling, jointing, silking, filling, and maturity stages, respectively. Changes in the leaf area index varied across different growth stages. At the seedling stage, the leaf area index showed a continuous upward trend with increasing planting density. From M2 to M5, the leaf area index under chemical regulation and conventional planting patterns increased by 13.53~34.05% and 6.46~21.30%, respectively, compared with M1, with the most pronounced advantage observed at the M5 planting density. From the jointing stage to the filling stage, the leaf area index exhibited a trend of first increasing and then decreasing with increasing planting density. From M2 to M5, the leaf area index under chemical regulation and conventional planting patterns increased by 5.54~19.08%, 7.82~16.32%, 11.93~19.20%, 8.06~19.46%, 11.06~21.37% and 11.75~23.53%, respectively, compared with M1. The optimal planting densities for leaf growth at the jointing, silking, and filling stages were M4, M3 and M4, respectively. At the maturity stage, the leaf area index also exhibited a trend of first increasing and then decreasing with increasing planting density, with the advantage observed at M2. Under chemical regulation and conventional planting patterns, compared with M1, the leaf area index at M2 and M3 increased by 6.65% and 5.76% (chemical regulation) and by 1.32% and 0.79% (conventional planting), respectively, whereas at M4 and M5, it decreased by 3.33% and 4.06% (chemical regulation) and by 9.24% and 9.42% (conventional planting), respectively. It is evident that appropriate planting density combined with chemical regulation measures can significantly increase the leaf area index of grain maize across all growth stages. Although overcrowded planting results in a higher leaf area index at early stages, it tends to cause premature leaf senescence, leading to a rapid decline in the leaf area index during the late filling stage and thus failing to maintain a sustained advantage.

### 2.3. Leaf Structural Parameters

**Figure 3:**
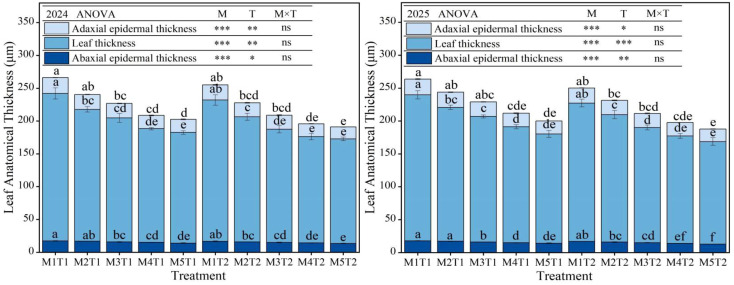
Effect of planting density and chemical regulation on leaf anatomical structure of grain maize. Bars and error bars stand to represent averaged values ± standard errors (n = 3), different lowercase letters indicate significant differences among the treatments (*p* < 0.05), and *, ** and *** indicate significant differences among the different treatments at the levels of *p* < 0.05, *p* < 0.01 and *p* < 0.001. ns means not significant at the level of *p* ≥ 0.05.

Planting density and chemical regulating measures had a significant effect on the thickness of the adaxial epidermis, abaxial epidermis and leaf of grain maize, and exhibited similar variation patterns across the experiment years. Analysis of variance revealed that planting density and chemical regulation measures had significant (*p* < 0.05) or highly significant effects (*p* < 0.01) on all indicators of maize leaf thickness, whereas their interaction had no significant effect (*p* > 0.05) (Figure 3), indicating that the regulatory effects of planting density and chemical regulation measures on maize leaf structure are also relatively independent. An analysis of the average data from the two growing seasons revealed that, under the same planting density, chemical regulation measures increased the thickness of adaxial epidermis thickness, abaxial epidermis thickness and leaf thickness. Specifically, the increase in adaxial epidermis thickness ranged from 1.92~6.40%, whilst the increase in abaxial epidermis thickness ranged from 4.58~7.26%, and leaf thickness by 4.93~9.25%, although these increases did not differ significantly. Under both chemical regulation measures and conventional planting conditions, as planting density increased, all indicators of grain maize leaf thickness exhibited a continuous decreasing trend. Compared with M1, the adaxial epidermis thickness of maize at M2 and M3 decreased by only 4.93% and 8.22% (chemical regulation) and 6.49% and 6.97% (conventional planting), respectively, with no significant difference, whereas the reductions at M4 and M5 reached 15.12% and 17.47% (chemical regulation) and 12.84% and 18.72% (conventional planting), respectively. The abaxial epidermis thickness of maize at M2 decreased by only 3.07% and 5.50%, with no significant difference, whereas the reductions at M3 to M5 ranged from 9.24~19.72% and 10.64~21.72%, respectively. For leaf thickness, the reductions ranged from 9.45~25.08% and 9.71~25.97%, respectively. Overall, high-density planting significantly reduced maize leaf thickness and epidermal tissue thickness, which is beneficial for improving light penetration in the middle and lower canopy layers; however, it may have potential effects on the accumulation of photosynthetic products and stress tolerance. In contrast, chemical regulation measures increased leaf thickness and epidermal tissue thickness across all planting density levels, indicating that while optimizing plant architecture, chemical regulation can maintain or enhance leaf tissue structure, thereby helping to preserve leaf photosynthetic function and stress tolerance under high-density planting conditions.

### 2.4. Stem Diameter

**Figure 4:**
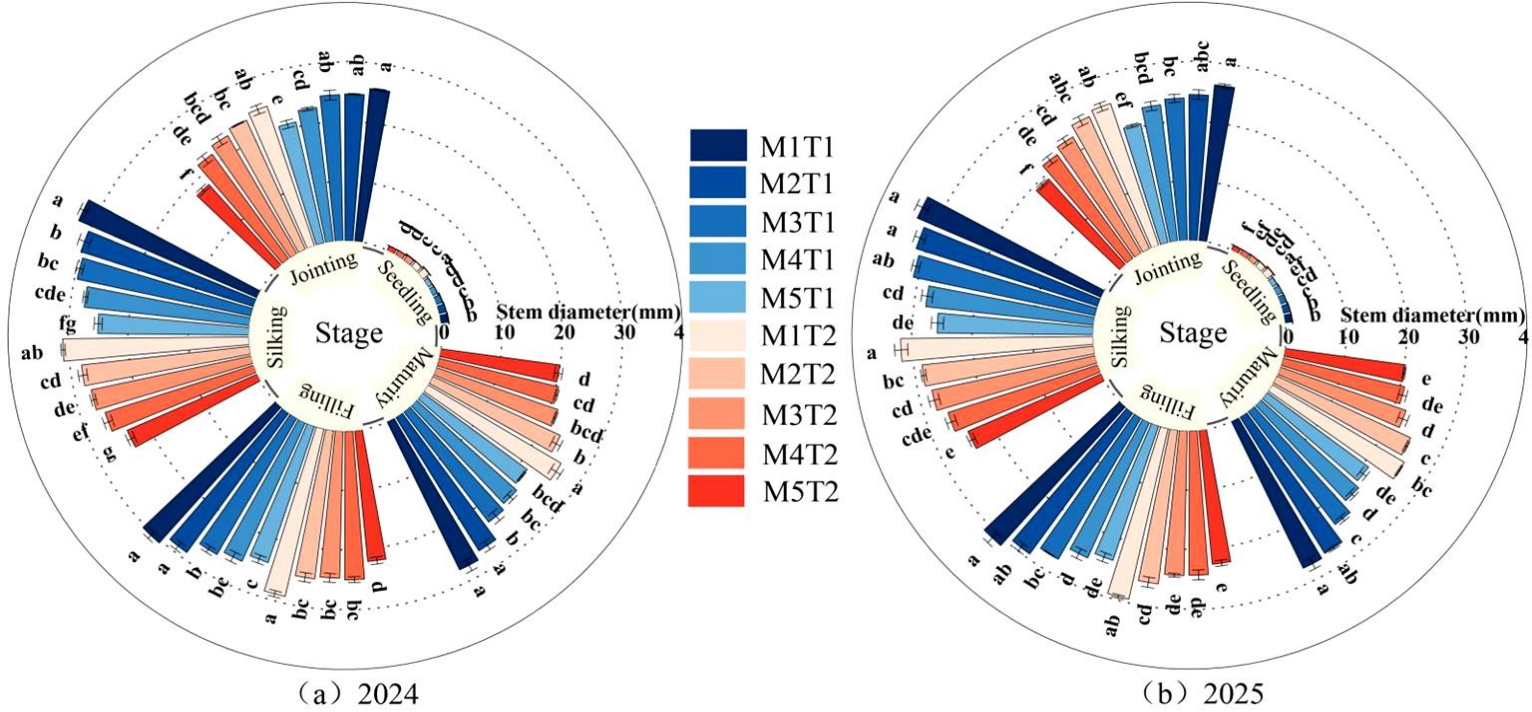
Effect of planting density and chemical regulation on stem diameter of grain maize. Bars and error bars stand to represent averaged values ± standard errors (n = 3); different lowercase letters indicate significant differences among the treatments (*p* < 0.05).

The stem diameter of grain maize plant increased rapidly from the jointing stage to the silking stage, reaching a maximum at the silking stage and then decreasing slightly thereafter (Figure 4). Different planting density and chemical regulation treatments had a significant effect on the stem diameter of grain maize. Analysis of variance revealed that planting density had a highly significant effect (*p* < 0.01) on stem diameter at all growth stages of grain maize, whilst the chemical regulation measure had a significant (*p* < 0.05) or highly significant effect on stem diameter from the jointing stage to maturity stage. However, their interaction was not significant (*p* > 0.05) (Table 2). An analysis of the average data from the two growing seasons revealed that, under the same planting density, chemical regulation measures effectively increased the stem diameter of grain maize compared with the conventional planting pattern, with increases of 5.33~11.16%, 2.12~11.04%, 7.02~10.06%, 2.71~13.79%, and 6.29~11.24% at the seedling, jointing, silking, filling, and maturity stages, respectively. The stem diameter exhibited a continuous decreasing trend with increasing planting density across all growth stages. Specifically, stem diameter was greatest at M1 and smallest at M5. Under both chemical regulation measures and conventional planting conditions, compared with M1, stem diameter from M2 to M5 was reduced by 11.70~36.88%, 5.59~20.53%, 5.87~22.33%, 5.73~22.18%, and 3.16~22.55% (chemical regulation) and 13.51~34.75%, 3.41~25.22%, 9.75~24.22%, 12.30~23.34% and 6.50~21.73% (conventional planting), respectively. Notably, under the chemical regulation measures, the reduction in stem diameter at M2 was smaller from the jointing stage to the maturity stage, and no significant difference was observed compared with M1. Evidently, overcrowded planting inhibits the growth of individual stem diameters. However, chemical regulation measures, by modulating endogenous hormone levels, effectively inhibit excessive internode elongation during the vegetative growth stage and promote stem thickening (transverse growth), thereby enhancing stem mechanical strength and improving lodging resistance.

### 2.5. Stem Mechanical Indicators

Planting density and chemical regulation measures exert significant regulatory effects on the stem mechanical properties of grain maize. Analysis of variance revealed that both planting density and chemical regulation measures had a highly significant effect on the stem breaking resistance and rind puncture strength of maize (*p* < 0.01), whereas their interaction had no significant effect (*p* > 0.05) (Table 3). An analysis of the average data from the two growing seasons revealed that, under the same planting density, chemical regulation measures effectively increased the stem breaking strength and rind puncture strength compared with conventional planting patterns, with the increase in magnitude depending on planting density. At the silking stage, both the stem breaking resistance and rind puncture strength of grain maize exhibited a continuous decreasing trend with increasing planting density. Under both chemical regulation measures and conventional planting conditions, there was no significant difference in stem breaking resistance and rind puncture strength between M2 and M1, indicating that appropriate increases in planting density did not significantly affect stem strength. However, M3, M4 and M5 showed significant reductions of 15.55~34.92%, 15.27~28.67%, 18.56~36.29% and 13.22~30.40%, respectively, compared with M1. Chemical regulation measures can effectively mitigate the negative effects of high planting density on stem mechanical properties. Under chemical regulation, stem breaking resistance increased by 5.98~10.92%, and rind puncture strength by 4.84~12.12%, compared with conventional planting. At 15 days after silking, the stem breaking resistance and rind puncture strength under chemical regulation measures increased by 2.69~13.56% and 4.87~12.69%, respectively, compared with conventional planting. Under different planting densities, there were no significant differences in stem breaking resistance and rind puncture strength between M2 and M1 under both chemical regulation and conventional planting conditions. However, M3, M4 and M5 showed significant reductions of 19.04~37.24%, 16.03~29.82% and 22.15~37.84%, and 19.33~29.72%, respectively, compared with M1. At 30 days after silking, the stem breaking resistance and rind puncture strength under chemical regulation measures increased by 7.71~23.11% and 5.17~12.65%, respectively, compared with conventional planting. Under different planting densities, there was still no significant difference in stem breaking resistance and rind puncture strength between M2 and M1 under both chemical regulation and conventional planting conditions. However, M3, M4 and M5 showed significant reductions of 23.90~35.93%, 15.94~31.74% and 21.28~37.26%, and 16.92~29.74%, respectively, compared with M1. Meanwhile, as the growth stage progressed, the stem breaking resistance of maize under all treatments exhibited a continuous decreasing trend, whereas rind puncture strength showed a trend of first increasing and then decreasing, reaching a peak at 15 days after the silking stage. Compared with the silking stage, the stem breaking strength across all treatments decreased by 27.74~40.70% at 30 days after the silking stage, whilst the rind puncture strength showed little change. Overall, although increasing planting density is beneficial for constructing an efficient canopy, planting density exceeding 112,500 plants·ha^−1^ significantly impairs stem mechanical properties and increases the risk of lodging at later stages. However, chemical regulation measures can effectively improve stem traits, enhance stem mechanical strength and increase stem lodging resistance.

### 2.6. Stem Breakage Rate

**Figure 5:**
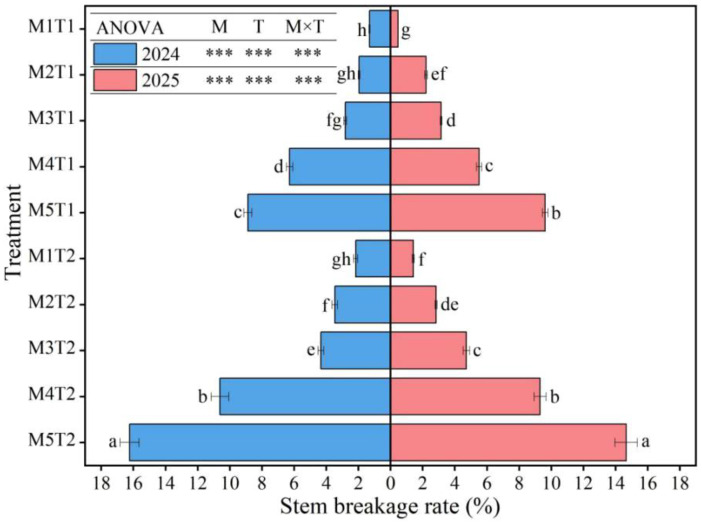
Effect of planting density and chemical regulation on stem breakage rate of grain maize. Bars and error bars stand to represent averaged values ± standard errors (n = 3), different lowercase letters indicate significant differences among the treatments (*p* < 0.05), and *** indicates significant differences among the different treatments at the level of *p* < 0.001.

As shown in Figure 5, planting density, chemical regulation measures and their interaction all had a highly significant effect on the stem breakage rate (*p* < 0.01). An analysis of the average data from the two growing seasons revealed that, with increasing planting density, the stem breakage rate of grain maize exhibited a continuous increasing trend. Under both chemical regulation and conventional planting conditions, compared with M1, the stem breakage rate at M2, M3, M4, and M5 increased significantly by 135.03%, 236.72%, 566.67%, and 944.63% (chemical regulation), and by 75.98%, 153.07%, 456.42%, and 763.13% (conventional planting), respectively. At the same planting density, chemical regulation measures significantly reduced the stem breakage rate of grain maize by 33.97~50.56% compared with conventional planting. Therefore, it can be concluded that increasing planting density significantly raises the stem breakage rate of grain maize; in particular, the risk of stem breakage rises sharply beyond the M3 density threshold, directly constraining the realization of yield potential under high-density planting. Chemical regulation measures can effectively reduce the stem breakage rate at all density levels, and there is a highly significant interaction effect with planting density, which can significantly enhance the lodging resistance of maize under high-density planting conditions.

### 2.7. Dry Matter Accumulation

**Figure 6:**
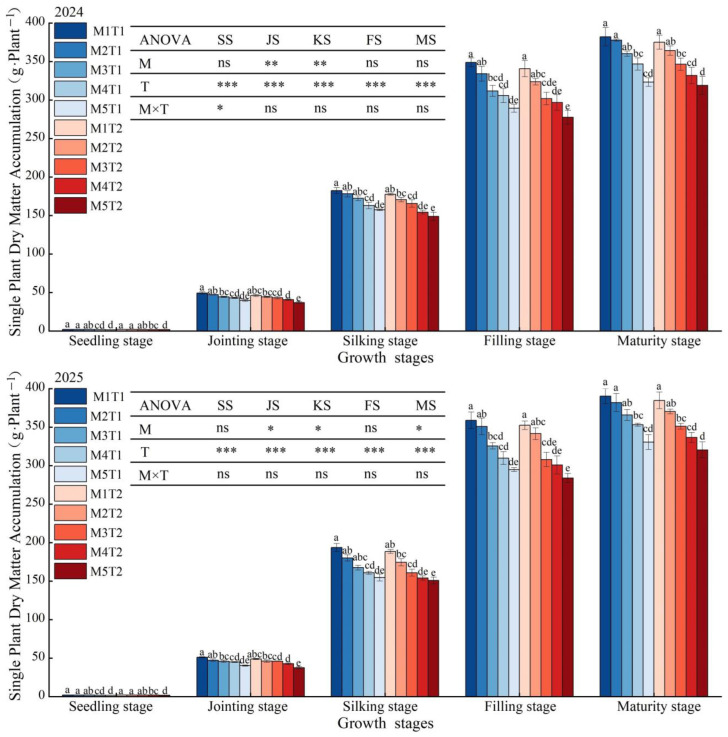
Effect of planting density and chemical regulation on dry matter accumulation of grain maize. Bars and error bars stand to represent averaged values ± standard errors (n = 3); different lowercase letters indicate significant differences among the treatments (*p* < 0.05); and *, ** and *** indicate significant differences among the different treatments at the levels of *p* < 0.05, *p* < 0.01 and *p* < 0.001. ns means not significant at the level of *p* ≥ 0.05. SS, seedling stage; JS, jointing stage; KS, silking stage; FS, filling stage; MS, maturity stage.

The dry matter accumulation per plant of grain maize showed a continuous increasing trend as the growth stages progressed, reaching a maximum at the maturity stage. Furthermore, different planting density and chemical regulation treatments had a significant effect on dry matter accumulation (Figure 6). Analysis of variance revealed that planting density had a highly significant effect (*p* < 0.01) on dry matter accumulation at all growth stages. Chemical regulation measures had a significant (*p* < 0.05) or highly significant (*p* < 0.01) effect on dry matter accumulation only at the jointing and silking stages. However, the interaction between the two factors was not significant at any growth stages (*p* > 0.05). An analysis of the average data from the two growing seasons revealed that, under the same planting density, the chemical regulation treatments increased dry matter accumulation compared with conventional planting patterns, with increases of 1.24~2.39%, 1.27~7.48%, 2.69~5.05%, 2.12~4.52%, and 1.65~4.72% at the seedling, jointing, silking, filling, and maturity stages, respectively. However, the increases were relatively small and not significant. At all growth stages, dry matter accumulation showed a consistent decreasing trend as planting density increased, with the most significant inhibition of dry matter accumulation per plant observed at the M5 planting density. Under both chemical regulation measures and conventional planting conditions, compared with M1, at planting densities M2 to M5, the dry matter accumulation per plant at the seedling, jointing, silking, filling and maturity stages were reduced by 3.92~17.89%, 5.89~20.44%, 4.69~17.02%, 3.20~17.47%, and 1.64~15.35%, and 3.72~13.40%, 4.75~21.69%, 5.79~18.09%, 4.02~18.97%, and 3.34~15.84%, respectively. Under chemical regulation measures at the maturity stage, the reductions at M2 and M3 were relatively small, and there was no significant difference compared with M1. It is evident that overcrowded planting is detrimental to the accumulation of dry matter per plant. Specifically, when the planting density exceeds the M3 threshold, dry matter accumulation per plant decreases significantly. Chemical regulation measures can effectively alleviate competitive pressure in high-density planting conditions and promote dry matter accumulation, with the effects being particularly pronounced at M2 and M3.

### 2.8. Yield Component

The effects of different planting densities and chemical regulation measures on the yield components of grain maize varied (Table 4). Analysis of variance indicated that planting density had a highly significant (*p* < 0.01) or significant (*p* < 0.05) effect on ear length, barren ear length, ear diameter, kernel number per ear, ear weight, kernel weight and 100-kernel weight. In contrast, chemical regulation measures had significant effects only on ear length and kernel weight. Furthermore, the interaction between the two factors was only highly significant for barren ear length, while it had no significant effect on other yield components (*p* > 0.05). An analysis of the average data from the two growing seasons revealed that, although the chemical regulation measures slightly improved the yield components of grain maize, there was no significant difference. As planting density increased, ear length exhibited a trend of first increasing and then decreasing. M3T1 and M3T2 produced the longest ears, which were 4.12% and 10.22% longer than those under M1T1 and M1T2, respectively. In contrast, M5T1 and M5T2 produced the shortest ears, which were 8.24% and 5.11% shorter than those under M1T1 and M1T2, respectively. There were no significant changes among the other treatments. The barren ear length exhibited a trend of first increasing and then decreasing with increasing planting density. M3T1 and M3T2 produced the shortest barren ear lengths, followed by M2T1 and M2T2, which were significantly reduced by 36.67% and 37.14%, and 10.00% and 25.71%, respectively, compared with M1T1 and M1T2. In contrast, M5T1 and M5T2 had the longest barren ear lengths, which were significantly increased by 20.00% and 34.29%, respectively, compared with M1T1 and M1T2. The ear diameter exhibited a continuous decreasing trend with increasing planting density. The smallest ear diameters were observed under M5T1 and M5T2, reduced by 10.97% and 6.76%, respectively, compared with M1T1 and M1T2. No significant differences were observed among the other treatments. The kernel number per ear, ear weight and kernel weight all exhibited a trend of first increasing and then decreasing with increasing planting density, with maximum and minimum values recorded at M3 and M5, respectively. The increases under M3T1 and M3T2 were 9.57%, 5.80%, and 5.05% (M3T1), and 13.40%, 1.85%, and 8.29% (M3T2), respectively. In contrast, the decreases under M5T1 and M5T2 were 8.96%, 27.20%, and 27.80% (M5T1), and 4.47%, 27.87%, and 26.24% (M5T2), respectively. With increasing planting density, 100-kernel weight exhibited a decreasing trend. However, there was no significant difference between the M2 or M3 treatments and M1, whilst the M4 and M5 treatments showed a significant decrease, ranging from 10.88~11.22% and 20.67~20.68%, respectively. It is evident that planting density and chemical regulation measures synergistically regulate ear development and kernel filling, thereby jointly influencing the formation of yield components. Under chemical regulation measures, the M3 treatment optimized ear structure and increased kernel number per ear and kernel weight, thereby enhancing productivity per plant. However, excessive planting density was detrimental to ear trait formation and kernel development, leading to a reduction in kernel weight.

### 2.9. Yield and Dry Matter Accumulation

**Figure 7:**
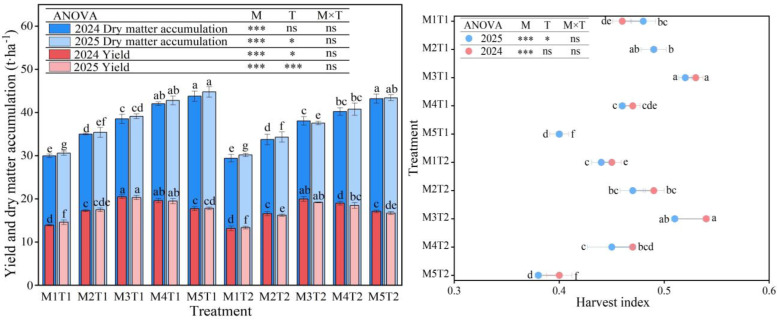
Effect of planting density and chemical regulation on yield, dry matter accumulation and harvest index of grain maize. Bars and error bars stand to represent averaged values ± standard errors (n = 3), different lowercase letters indicate significant differences among the treatments (*p* < 0.05); * and *** indicate significant differences among the different treatments at the levels of *p* < 0.05 and *p* < 0.001. ns means not significant at the level of *p* ≥ 0.05.

The results of the analysis of variance shown in Figure 7 indicate that planting density has a highly significant effect on yield, dry matter accumulation and harvest index (*p* < 0.01). Chemical regulation measures had a significant (*p* < 0.05) or highly significant effect only on yield, whilst the interaction between the two factors had no significant effect (*p* > 0.05). Meanwhile, it was found that with increasing planting density, the yield and harvest index of spring corn showed a trend of first increasing and then decreasing, whereas the dry matter accumulation continued to increase. Based on an analysis of the average data from the two growing seasons, under both the chemical regulation measures and conventional planting conditions, the yields at M2, M3, M4 and M5 were significantly higher than those at M1 by 19.20%, 34.53%, 40.49% and 40.23% (chemical regulation), and 18.22%, 33.96%, 39.06% and 41.62% (conventional planting), respectively. Meanwhile, dry matter accumulation was significantly higher than that of M1 by 17.00%, 31.48%, 37.82% and 38.47% (chemical regulation), and 16.05%, 30.34%, 36.11% and 38.08% (conventional planting), respectively. It can be seen that yield peaked at the M3 density; when the density exceeded M3, yield began to decline. Therefore, the M3 density is the threshold for achieving high yield. In contrast, dry matter accumulation reached its maximum at the M5 density. Among all treatments, M3T1 and M3T2 yielded the highest harvest indices, both reaching 0.53. However, as density increased to M5, the harvest index decreased significantly by 12.36~14.89%, compared with M1. This indicates that once planting density exceeded the M3 threshold, the vegetative growth became excessive while the reproductive allocation was insufficient, resulting in a decrease in the harvest index. Under the same planting density, although chemical regulation measures increased grain maize yield, dry matter accumulation and the harvest index compared with the conventional planting patterns, the increases were only 1.99~4.06%, 3.80~5.11% and 1.09~5.62%, respectively, and there were no significant differences. It is evident that high-density planting significantly influences the yield formation and dry matter allocation of grain maize. In particular, once the planting density exceeds the M3 threshold, although dry matter accumulation continues to increase, yield continues to decrease. However, chemical regulation measures can effectively improve dry matter accumulation and allocation, enhance the harvest index, and increase yield potential.

## 3. Discussion

Chemical regulation is a primary measure for coordinating the growth between population and individual plants under high-density planting, thereby preventing lodging and achieving high yield [30]. Overcrowded planting leads to canopy closure, triggering a shading response in the plants, which results in excessive internode elongation and poor development of mechanical tissues, thereby increasing the risk of stem breakage at later stages. Chemical regulation measures, by regulating the balance of endogenous hormones, have been shown to inhibit internode elongation and improve stem mechanical strength [31]. The results of this study indicated that, with increasing planting density, the stem breaking resistance and rind puncture strength of grain maize exhibited a continuous decreasing trend, and the reduction became significant when the planting density exceeded 112,500 plants·ha^−1^. Spraying chemical regulators significantly increased stem breaking resistance and rind puncture strength at 30 days after the silking stage, with increases ranging from 7.71~23.11% and 5.17~12.65%, respectively, thereby effectively mitigating the lodging effects that may result from high-density planting. Zhang et al. [32] reported that with increasing planting density, the number and area of large vascular bundles per unit area of maize stem decreased, and the degree of lignification of parenchyma cells was reduced, leading to a significant reduction in stem puncture strength and breaking resistance, which is consistent with the findings of the present study. Liu et al. [33] pointed out that chemical regulation measures significantly reduced the length of the basal internodes of maize by inhibiting gibberellin biosynthesis, increased stem diameter and wall thickness, and consequently enhanced the compressive and bending resistance of the stem. This study further confirmed the former findings, demonstrating that the stem mechanical indicators of grain maize were significantly improved after spraying chemical regulators, indicating that chemical regulation measures can enhance physical strength by optimizing the stem’s morphological structure. However, this study found that when spraying chemical regulators under the high-density planting condition of 142,500 plants·ha^−1^, the stem breaking resistance remained significantly lower than that under conventional low-density planting of 82,500 plants·ha^−1^, which is slightly different from the viewpoints of some scholars [34]. This may be due to the fact that, once the density exceeds the threshold of 112,500 plants·ha^−1^, light intensity within the canopy decreases, leading to insufficient supply of photosynthetic products. Although exogenous hormones inhibit internode elongation, the allocation of carbohydrates required for the development of vascular bundles and sclerenchyma tissues in the stems is restricted, thereby preventing the ‘chemical regulation-induced robustness’ effect from fully mitigating the ‘slender stem’ effect caused by high-density planting. Moreover, the availability of soil nutrients, as influenced by fertilization regimes, also plays a critical role in stem mechanical strength. Symanowicz et al. [35] reported that the application of NPKMgS fertilizers combined with low-energy lignite significantly increased the uptake of nitrogen, phosphorus, and potassium in maize, which are essential for stem lignin synthesis and structural integrity. Similarly, Symanowicz et al. [36] found that such fertilization practices enhanced soil urease, phosphatase, and dehydrogenase activities, thereby promoting overall plant vigor and stress tolerance. Therefore, optimizing both planting density and soil nutrient management may synergistically enhance lodging resistance. Furthermore, different varieties exhibit varying degrees of sensitivity to chemical regulators, which may also have a certain influence on the performance of mechanical properties [37]. Meanwhile, this study found that at 30 days after the silking stage, the stem breaking resistance had decreased by 27.74~40.70% compared with the silking stage. However, the magnitude of decline under high planting densities (142,500 and 127,500 plants·ha^−1^) was significantly greater than that under low and medium densities. This indicates that excessive planting density intensifies ‘source–sink’ competition, leading to reduced stem plumpness and the deterioration of mechanical support functions. However, chemical regulation measures were still able to maintain high mechanical strength at 30 days after the silking stage, and the stem breakage rate was significantly lower than that under the conventional planting pattern. This indicates that chemical regulators not only improve stem structure but also provide energy substance support for the stem by delaying leaf senescence and maintaining photosynthetic function during the filling stage. Consequently, in the Hexi Oasis irrigation area, controlling the planting density of 112,500 plants·ha^−1^ combined with chemical regulation can maintain stem mechanical properties and enhance plant lodging resistance by optimizing stem morphological structure and dry matter allocation.

The leaf is the primary organ of crop photosynthesis, and its size and functional duration directly determine light interception capacity and dry matter production potential [38]. In this study, the leaf area index followed a unimodal curve with increasing planting density, reaching a maximum at the silking stage. However, under high planting densities of 127,500 and 142,500 plants·ha^−1^, the leaf area index declined rapidly during the late filling stage, with a significantly greater decline compared with that of medium and low densities. This is consistent with the findings of Wang et al. [39], who suggested that under high-density populations, light intensity of the middle and lower leaves during the late growth stage falls below the light compensation point for extended periods, leading to premature leaf senescence. By analyzing the anatomical structure of the leaves, it was found that with increasing planting density, the thickness of adaxial epidermis, abaxial epidermis and leaf all showed a continuous decreasing trend. Under the high planting density of 142,500 plants·ha^−1^, leaf thickness decreased by 25.08~25.97% compared with that under 82,500 plants·ha^−1^, indicating that high-density planting induces poor development of leaf tissue. This conclusion is consistent with the findings of Yang et al. [40], which may be attributed to the impaired development of the palisade and spongy tissues under high-density planting conditions. Meanwhile, the results of this study revealed that chemical regulation measures could significantly increase leaf thickness and epidermal tissue thickness, with an increase ranging from 4.93~9.25%. This conclusion was also confirmed in the study by Ali et al. [41]. This might be due to the fact that aminoethyl hexanoate·ethephon induces an increase in cytokinin content in leaves, thereby delaying leaf senescence and promoting cell division and expansion. Therefore, chemical regulation measures can simultaneously prolong the leaf functional period under high-density planting conditions, providing a sustained photosynthetic source for dry matter accumulation during the middle and late growth stages. However, this study did not quantitatively analyze endogenous hormones (such as cytokinins, gibberellins, or auxins) in the tissues of maize plants, and thus the explanation could only be based on the conclusions of previous studies. In later experiments, the dynamic changes in endogenous hormones will be directly measured to more accurately clarify the physiological mechanism by which chemical regulation modulates stem and leaf development under different planting densities.

Stem diameter is a core indicator of individual plant robustness in maize [42]. In this experiment, stem diameter continuously decreased with increasing planting density, and from the jointing stage to the maturity stage, the reduction at the high density of 142,500 plants·ha^−1^ reached 20.53~36.88% compared with the low density of 82,500 plants·ha^−1^. This finding is similar to the conclusion of Shi et al. [43], who suggested that under high-density planting conditions, intensified competition among plants leads to preferential allocation of photosynthetic products to vertical elongation rather than to increases in transverse diameter, resulting in slender and weak stems. Yu et al. [44] reported that the combined application of aminoethyl hexanoate·ethephon inhibits gibberellin synthesis while promoting the synergistic action of cytokinins and auxins, which facilitates cell division and expansion in internodes and promotes transverse growth of the stem. The results of this study further confirm the former conclusion, indicating that chemical regulation can significantly increase stem diameter, with increases ranging from 2.12~13.79%. This is consistent with the findings of Liu et al. [45,46], who reported that under high-density planting conditions, the application of chemical regulators increased the lignin, cellulose and hemicellulose contents at the base of the third internode of maize, effectively increasing stem diameter. However, the magnitude of increase varied slightly, which may be attributed to differences in planting patterns or variations in the density tolerance of the tested varieties.

As influential factors of population yield in maize, planting density and chemical regulation measures are key agronomic practices regulating yield formation. Yield components such as ear traits, kernel number per ear and 100-kernel weight are all influenced by planting density and chemical regulation measures [47,48]. The planting density alters the nutritional space of individual plants and the light interception rate of the canopy, thereby affecting the establishment of the kernel sink capacity. Meanwhile, chemical regulation measures effectively alleviate the stress caused by high-density planting by regulating the balance of endogenous hormones and the efficiency of stem transportation. The interaction effect of the two factors jointly influences the formation of yield components [49,50]. In this experiment, with increasing planting density, the ear length, ear diameter, kernel number per ear, and 100-kernel weight of maize all exhibited a decreasing trend. However, the number of ears per unit area increased, exhibiting a yield compensation relationship. Among them, under the synergistic effect of a planting density of 112,500 plants·ha^−1^ and chemical regulation measures, the maize ear traits were optimal, with the highest kernel number per ear and higher 100-kernel weight. This was because the interaction between high-density planting and chemical regulation optimized the canopy structure of the population and increased the individual light interception rate. Meanwhile, chemical regulation improved stem translocation efficiency, promoting dry matter transport to grains during the silking stage, thereby enhancing grain sink capacity and accumulation. This is consistent with the conclusion of Shi et al. [51], indicating that under an appropriate planting density, canopy light distribution is optimized, and there is sufficient photosynthetic product supply after flowering, which can meet the grain filling requirements. However, when the planting density exceeds 112,500 plants·ha^−1^, kernel number per ear and 100-kernel weight decreased significantly, while barren ear tip length increased significantly. This indicates that excessive density planting will lead to mutual shading among adjacent plants, restricting the interception and utilization efficiency of light by individual plants. As a result, apical kernel abortion occurs, and plants may even exhibit premature senescence and yield reduction. This conclusion was also verified in the study by Chen et al. [52].

Planting density and chemical regulation measures jointly determine the final kernel yield by regulating yield components and dry matter accumulation and allocation [53]. The results of this study indicated that the yield of grain maize showed a trend of first increasing and then decreasing with the increase in planting density. Among them, the yield was the highest at a planting density of 112,500 plants·ha^−1^, and after exceeding this threshold, the yield began to decline. Although spraying chemical regulators showed a yield-increasing effect compared with conventional planting patterns, the increase was only 1.99~4.06%, indicating that planting density is the primary factor determining yield. The magnitude of yield increase resulting from chemical regulation measures did not change significantly. However, research by Huang et al. [54] suggested that spraying chemical regulators could significantly increase maize yield under high-density planting by shortening internodes, enhancing lodging resistance and promoting photosynthesis. This discrepancy with the findings of this study may be due to differences in the maize varieties tested, or variations in experimental methods or geographical locations, which may have led to differing results. Furthermore, this study also found that the harvest index showed a trend of first increasing and then decreasing with increasing planting density, while population dry matter accumulation continued to increase. This indicates that excessive planting density leads to excessive vegetative growth and insufficient reproductive allocation, resulting in a yield-limiting pattern of ‘sufficient source but weak sink’. Research by Cao et al. [55] indicated that under high-density planting conditions, the proportion of dry matter accumulated between the silking and maturity stages that is allocated to the kernel is significantly reduced, resulting in a marked decline in yield. The findings of this study further confirm the above conclusion. Under the high planting density of 142,500 plants·ha^−1^, the population dry matter accumulation reached its highest level, but the harvest index was significantly lower than that of the low density of 82,500 plants·ha^−1^, with a reduction of 12.36~14.89%, resulting in an imbalance in the source–sink relationship characterized by ‘high biomass but low harvest index’. This contradiction between increased total biomass and decreased kernel yield highlights the source–sink imbalance under ultra-high-density conditions. Although the photosynthetic capacity of the population remains at a high level, the ability of individual grains to receive and utilize assimilates is limited, resulting in a reduction in kernel number per ear, a decrease in kernel weight, and an increase in barren ear length when the density exceeds 112,500 plants·ha^−1^. However, in this study, the kernel number per leaf area ratio and the dry matter remobilization rate during filling stage were not calculated. Therefore, future research should systematically evaluate source–sink indicators, including the reutilization rate of non-structural carbon compounds, the contribution of post-silking dry matter accumulation to kernel yield, and the ratio of leaf area to kernel, to elucidate the reasons for the phenomenon of ‘high biomass but low harvest index’ caused by increased planting density. Consequently, an appropriate planting density combined with chemical regulators can achieve the synchronization of population structure optimization and individual development coordination, thereby achieving the objective of increasing yields through high-density planting coupled with chemical regulation. Among all treatments, M3T1 yielded the greatest increase in yield.

## 4. Materials and Methods

### 4.1. Experimental Site Profile

**Figure 8:**
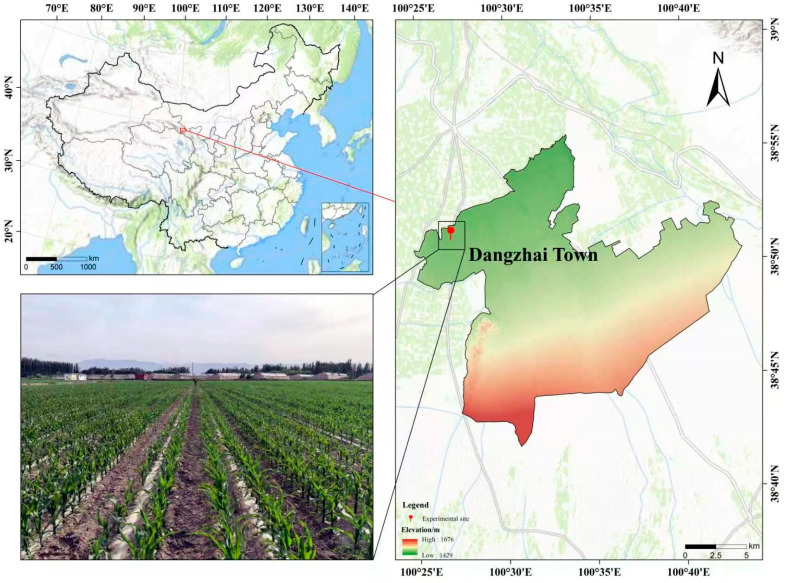
Location of the experimental site.

**Figure 9:**
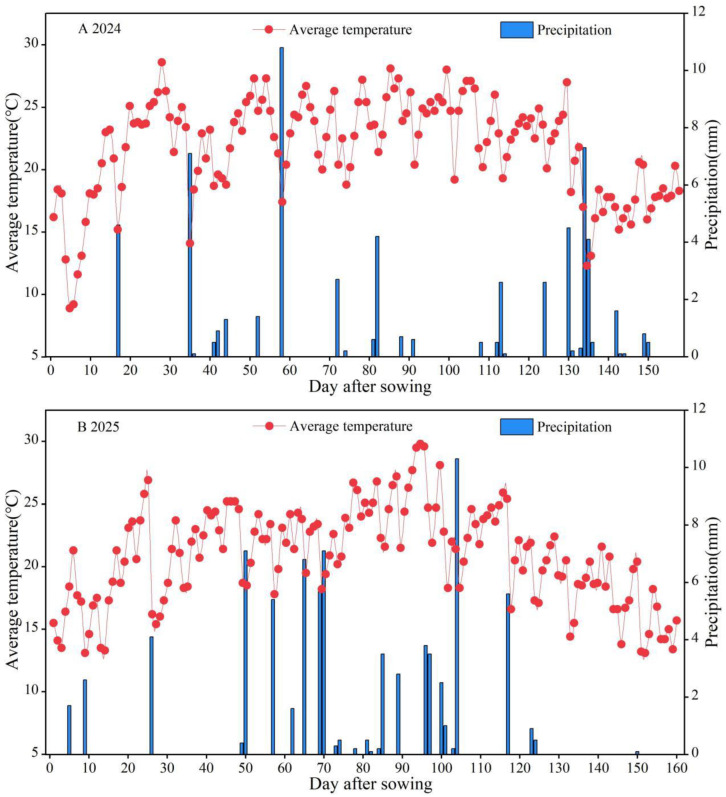
Daily variation in average temperature and precipitation throughout maize growing seasons of 2024 (**A**) and 2025 (**B**).

The experimental site is located in Dangzhai Town, Zhangye City, Gansu Province (100°28′ E, 38°49′ N, 1462 m a.s.l.) (Figure 8). The experimental area has a temperate continental arid climate, with an altitude of approximately 1367 m. The average annual temperature is 7.3 °C, the effective accumulated temperature ≥ 10 °C is 2980 °C, the average annual sunshine duration is 3085 h, the frost-free period is 165 days, the average annual rainfall is 130 mm, and the annual evaporation is 1788 mm. The soil type is sandy loam. In the 0–20 cm soil layer, the particle size composition is as follows: 0.2~2.0 mm accounts for 17.5%, 0.02~0.2 mm accounts for 43.0%, 0.002~0.02 mm accounts for 27.1%, and <0.002 mm accounts for 12.5%. The basic soil nutrient comprises a pH in H_2_O of 8.47, organic matter of 13.1 g kg^−1^, total nitrogen of 0.75 g kg^−1^, alkali–hydrolysable nitrogen of 120.7 mg kg^−1^, available phosphorus of 30.2 mg kg^−1^, and available potassium of 516 mg kg^−1^, with a total water-soluble salt content of 3.3 g kg^−1^. Precipitation during the maize growing season was 62.0 mm in 2024 and 79.0 mm in 2025. The precipitation and average temperature over the experimental period are shown in Figure 9.

### 4.2. Experimental Materials and Design

#### Experimental Design

The experiment was conducted from April to September in 2024 and 2025. The grain maize variety tested was ‘Xianyu 1483’, a compact maize variety resistant to stem rot and ear rot. The experiment adopted a two-factor split-plot experimental design: planting density and chemical regulation treatment. The planting density was the main factor, with five density gradients: 82,500 plants·ha^−1^ (M1), 97,500 plants·ha^−1^ (M2), 112,500 plants·ha^−1^ (M3), 127,500 plants·ha^−1^ (M4) and 142,500 plants·ha^−1^ (M5). The conventional planting density in the local region ranges from 75,000~90,000 plants·ha^−1^. The five density gradients tested in this experiment ranged from 82,500~142,500 plants·ha^−1^, covering the local conventional density range and extending to higher densities to explore the effects of high-density planting. The secondary factor was the chemical regulation treatment: spraying 30% aminoethyl hexanoate·ethephon at the 10-leaf stage (T1) and spraying an equal amount of water as the control (T2). Spraying was carried out between 17:00 and 18:00 under sunny, windless weather conditions with no rainfall within 48 h. The spraying rate for each treatment was 300 mL·ha^−1^ [35]. The experiment consisted of 10 treatments; each treatment was replicated three times, arranged in a randomized block design. All plots were oriented east–west and measured 200 m^2^ (20.0 m × 10.0 m). All treatments were irrigated 11 times throughout the growing period. The application rates were 172.5 kg N·ha^−1^, 96.6 kg P·ha^−1^, and 46.8 kg K·ha^−1^. The nitrogen fertilizer was applied as top-dressing in a ratio of 4:3:3 at the jointing stage, the late whorl stage and the silking stage, respectively. Grain maize was continuously cropped for two years. In 2024, the experiment was sown on 24 April and harvested on 27 September. In 2025, the experiment was sown on 26 April and harvested on 1 October.

### 4.3. Measurement Items and Methods

#### 4.3.1. Plant Height, Ear Height and Ear-to-Plant Height Ratio

At the silking stage of maize, five plants with normal growth were randomly selected from each plot. Upon return to the laboratory, plant height and ear height were measured using a 5.0 m tape measure, and the ear-to-plant height ratio was calculated as follows: ear-to-plant height ratio = ear height/plant height.

#### 4.3.2. Growth Indicators

At the seedling, jointing, silking, filling and maturity stages of maize, three plants were randomly selected from each plot and taken back to the laboratory for measurement.

(1) Stem diameter

Stem diameter was measured using a vernier caliper with an accuracy of 0.01 mm. Measurements were taken at the base, the ear-bearing internode, and the first internode above the ear of the stem of each plant. The average of these three measurements was calculated as the stem diameter for that plot.

(2) Leaf area index and leaf structure

All leaves of each maize plant were cut at the junction of the leaf sheath and the leaf blade. The leaf area was measured using a YMJ-B leaf area meter (manufactured by Zhejiang Topu Yunnong Technology Co., Ltd., Hangzhou, China). The average leaf area was then calculated, and the leaf area index (LAI) was determined as LAI = total leaf area /land area. Meanwhile, at the silking stage, ear leaves measuring 1 cm × 2 cm were sampled from 1/3 of the distance from the base of the main vein. The samples were fixed in FAA fixative (90 mL of 70% anhydrous ethanol + 5 mL of formalin + 5 mL of glycerol + 5 mL of glacial acetic acid), stained with safranin-fast green, and then the adaxial epidermis thickness, abaxial epidermis thickness, and leaf thickness were measured using an MvImageView 3.0 image analysis system [56].

(3) Dry matter accumulation per plant

After the measurement of stem diameter and leaf area, the various plant organs were separated and placed on labeled trays. The samples were oven-dried at 105 °C for 2 h to deactivate enzymes, after which the temperature was reduced to 80 °C and drying continued until constant weight was achieved. The dry weight was measured using an electronic balance with an accuracy of 0.001 g (Delixi Electric Co., Ltd., Wenzhou, Zhejiang, China). The average values of the measurements were taken, and the aboveground dry matter accumulation was calculated.

#### 4.3.3. Determination of Stem Mechanical Indicators

At the silking stage, and at 15 and 30 days after silking, three representative maize plants were randomly selected from each plot. Using a YYD-1 stem strength tester (Zhejiang Top Instrument Co., Ltd., Hangzhou, China), a vertical thrust was gradually increased until the stem broke. The mechanical value recorded at breaking was taken as the stem breaking resistance. Meanwhile, the rind puncture strength at the third basal internode was measured using a probe with a cross-sectional area of 1 mm^2^. During the test, the probe was inserted slowly and uniformly, and the mechanical value required to penetrate the stem rind was recorded.

#### 4.3.4. Stem Breakage Rate

At maturity stage, the number of plants with broken internodes below the ear and the total number of plants in each plot were recorded. The stem breakage rate was calculated as follows: Stem breakage rate = (Number of plants with stem breakage/Total number of plants in the plot) × 100%.

#### 4.3.5. Yield and Yield Component

After maize maturity, yield measurements were taken from the middle three rows of each plot. The ears were sun-dried, threshed, and weighed. Meanwhile, 10 plants were randomly selected for drying to determine the accumulation of aboveground dry matter. Additionally, 20 ears were randomly selected from each plot to determine ear diameter, ear length, barren ear length, number of kernel rows, kernels per row, number of ears per plant, kernel number per ear, and 100-kernel weight. The harvest index was calculated as follows: kernel yield/aboveground dry matter accumulation.

### 4.4. Data Statistics and Analysis

Data processing was performed using Microsoft Excel (Version 2010, Microsoft Corp., Redmond, WA, USA) software. The statistical analysis and regression model establishment were performed by using the LSD multiple comparison method in SPSS 22.0 (IBM, Inc., New York, NY, USA) software, while the graphs were plotted using OriginPro 2025 (Origin Lab, Corp., Hampton, MA, USA).

## 5. Conclusions

Maintaining an appropriate planting density and implementing chemical regulation measures are scientific approaches to balancing the interests of individual plants and the population as a whole, and to reducing the risk of stress caused by adverse environmental conditions. This study indicates that planting density is the primary factor determining yield, whilst chemical regulation measures achieve synergistic benefits in high-density planting by optimizing plant morphology, enhancing lodging resistance, delaying leaf senescence and increasing the harvest index. However, as density continues to increase, the yield-enhancing effect gradually diminishes. Taking into account that both planting density and chemical regulation measures have significant main effects and can be applied in an additive manner to maize stem mechanical properties, leaf anatomical structure, dry matter accumulation and yield, it is recommended that a planting density of 112,500 plants·ha^−1^, together with spraying 30% aminoethyl hexanoate·ethephon at the 10-leaf stage, is the optimal approach for achieving high yield under high-density planting of grain maize in the Hexi Oasis irrigation area. This approach can effectively reduce plant height and ear height, enhance plant lodging resistance, and significantly increase both grain yield and dry matter accumulation. However, this study still has certain limitations. Firstly, in assessing stem lodging resistance, this study only measured stem breaking resistance, rind puncture strength and stem breakage rate. Future research should measure the length and wall thickness of basal internodes, the number and area of vascular bundles, and the contents of lignin and cellulose more core indicators of lodging resistance, to achieve a more comprehensive understanding of stem mechanical properties. Secondly, only one grain maize variety was tested in this experiment. Different maize varieties may exhibit significant differences in their responses to planting density and chemical regulation, as well as in their tolerance to high-density conditions. Therefore, multi-variety experiments should be conducted in future studies to validate the findings. Thirdly, this experiment applied only one chemical regulation dosage and did not test the effects of different dosages or concentrations on grain maize. Consequently, the optimal concentration and application rate of the plant growth regulator for grain maize under high-density planting conditions remain undetermined, which limits the precision of agricultural production guidance. In future research, addressing these limitations will more clearly elucidate the physiological and agronomic mechanisms by which chemical regulation improves lodging resistance and yield in high-density grain maize, thereby providing more reliable and practical suggestions for farmers and cooperatives in the Hexi Oasis irrigation area.

## Figures and Tables

**Table 1:** Results of variance analysis of planting density and chemical regulation on leaf area index of grain maize.

Year	Treatment	Seedling Stage	Jointing Stage	Silking Stage	Filling Stage	Maturity Stage
2024	Planting density (M)	***	***	***	***	***
	Chemical regulation (T)	ns	***	***	**	***
	M × T	*	ns	ns	ns	ns
2025	Planting density (M)	***	***	***	***	***
	Chemical regulation (T)	ns	***	***	**	***
	M × T	*	ns	ns	ns	ns

Note: *, **, and *** indicate significant differences among the different treatments at the levels of *p* < 0.05, *p* < 0.01, and *p* < 0.001, respectively. ns means not significant at the level of *p* ≥ 0.05.

**Table 2:** Results of variance analysis of planting density and chemical regulation on stem diameter of grain maize.

Year	Treatment	Seedling Stage	Jointing Stage	Silking Stage	Filling Stage	Maturity Stage
2024	Planting density (M)	***	***	***	***	***
	Chemical regulation (T)	ns	**	***	***	***
	M × T	ns	ns	ns	ns	ns
2025	Planting density (M)	***	***	***	***	***
	Chemical regulation (T)	ns	*	***	***	***
	M × T	ns	ns	ns	ns	ns

Note: *, **, and *** indicate significant differences among the different treatments at the levels of *p* < 0.05, *p* < 0.01, and *p* < 0.001, respectively. ns means not significant at the level of *p* ≥ 0.05.

**Table 3:** Effect of planting density and chemical regulation on stem mechanical indicators of grain maize.

Year	Treatment	Silking Stage		15 Days After Silking Stage		30 Days After Silking Stage	
		Breaking Resistance (N)	Rind Puncture Strength (N/mm^2^)	Breaking Resistance (N)	Rind Puncture Strength (N/mm^2^)	Breaking Resistance (N)	Rind Puncture Strength (N/mm^2^)
2024	M1T1	43.19 ± 1.42 a	56.48 ± 1.05 a	35.24 ± 0.73 a	65.92 ± 0.41 a	29.11 ± 1.07 a	59.17 ± 0.79 a
	M2T1	40.20 ± 0.49 b	53.07 ± 1.34 b	32.60 ± 1.00 bc	58.39 ± 1.63 bc	25.84 ± 0.63 b	56.32 ± 0.80 ab
	M3T1	35.51 ± 0.60 c	48.77 ± 0.97 cd	28.78 ± 0.48 d	54.47 ± 1.21 d	22.03 ± 0.57 d	50.49 ± 0.92 c
	M4T1	32.48 ± 1.23 d	44.12 ± 1.28 e	24.15 ± 0.70 e	49.09 ± 0.81 e	21.25 ± 0.34 d	45.26 ± 1.53 d
	M5T1	27.35 ± 0.36 ef	40.50 ± 0.77 f	22.00 ± 0.63 ef	45.55 ± 1.38 ef	18.76 ± 0.67 e	41.03 ± 1.32 e
	M1T2	40.27 ± 1.31 b	52.23 ± 1.14 b	33.81 ± 1.01 ab	59.44 ± 1.88 b	26.35 ± 0.60 b	54.76 ± 1.28 b
	M2T2	38.03 ± 0.75 bc	50.57 ± 1.24 bc	31.26 ± 1.10 c	55.16 ± 1.57 cd	24.02 ± 0.16 c	51.19 ± 1.37 c
	M3T2	32.15 ± 1.09 d	45.75 ± 1.43 de	26.68 ± 0.07 d	48.05 ± 1.31 e	20.70 ± 0.34 d	45.88 ± 1.35 d
	M4T2	29.99 ± 0.85 de	39.94 ± 0.48 f	21.84 ± 0.61 f	43.70 ± 0.75 fg	17.35 ± 0.59 e	41.20 ± 0.73 e
	M5T2	25.64 ± 0.91 f	36.51 ± 0.70 g	20.57 ± 0.35 f	41.28 ± 0.59 g	16.97 ± 0.58 e	39.51 ± 0.46 e
	ANOVA						
	Planting density (M)	80.35 ***	74.50 ***	118.50 ***	78.22 ***	91.13 ***	79.25 ***
	Chemical regulation (T)	17.29 ***	27.55 ***	13.75 **	42.91 ***	37.20 ***	31.66 ***
	M × T	0.22 ns	0.26 ns	0.19 ns	0.64 ns	1.45 ns	0.81 ns
2025	M1T1	41.75 ± 1.17 a	57.50 ± 1.77 a	36.80 ± 0.69 a	63.44 ± 2.09 a	30.17 ± 0.75 a	60.54 ± 0.81 a
	M2T1	39.95 ± 0.66 ab	55.08 ± 1.49 ab	33.02 ± 0.32 b	57.92 ± 1.06 bc	27.50 ± 0.78 b	58.65 ± 1.13 ab
	M3T1	36.22 ± 1.18 c	47.80 ± 1.35 cd	29.54 ± 0.92 c	54.16 ± 1.69 cd	23.08 ± 0.50 d	50.14 ± 1.02 de
	M4T1	31.61 ± 0.99 d	45.68 ± 1.66 d	25.68 ± 0.45 d	50.76 ± 0.47 de	22.01 ± 0.76 d	46.67 ± 0.35 ef
	M5T1	27.93 ± 0.35 e	40.80 ± 1.35 e	23.21 ± 0.73 e	45.24 ± 0.63 fg	19.22 ± 0.69 ef	40.68 ± 1.39 g
	M1T2	39.14 ± 1.29 ab	53.91 ± 1.65 ab	34.50 ± 0.93 b	60.06 ± 1.93 ab	26.85 ± 0.94 bc	55.81 ± 1.82 bc
	M2T2	37.60 ± 0.55 bc	51.41 ± 1.87 bc	32.64 ± 0.51 b	55.75 ± 1.38 c	25.26 ± 0.81 c	52.13 ± 1.81 cd
	M3T2	32.52 ± 0.36 d	46.36 ± 1.41 d	26.50 ± 0.84 d	48.35 ± 1.38 ef	21.18 ± 0.43 de	45.98 ± 1.36 f
	M4T2	30.00 ± 0.79 de	40.15 ± 0.64 e	22.04 ± 0.47 e	44.93 ± 1.17 fg	17.79 ± 0.59 fg	40.41 ± 1.10 g
	M5T2	24.95 ± 0.67 f	37.36 ± 0.25 e	21.89 ± 0.66 e	42.70 ± 0.65 g	16.41 ± 0.44 g	38.18 ± 1.12 g
	ANOVA						
	Planting density (M)	88.14 ***	47.11 ***	135.78 ***	54.64 ***	83.39 ***	77.70 ***
	Chemical regulation (T)	23.50 ***	15.29 **	24.39 ***	21.25 ***	44.31 ***	36.72 ***
	M × T	0.40 ns	0.51 ns	1.83 ns	0.85 ns	0.89 ns	0.85 ns

Note: Different lowercase letters in the same column indicate significant differences among the different treatments (*p* < 0.05). ** and *** indicate significant differences among the different treatments at the levels of *p* < 0.01 and *p* < 0.001, respectively. ns means not significant at the level of *p* ≥ 0.05.

**Table 4:** Effect of planting density and chemical regulation on yield component of grain maize.

Year	Treatment	Ear Length (cm)	Barren Ear Length (cm)	Ear Diameter (mm)	Kernel Number per Ear	Ear Weight (g)	Kernel Weight (g/plant)	100-Kernel Weight (g)
2024	M1T1	16.5 ± 0.33 bc	1.5 ± 0.05 cd	49.93 ± 1.46 ab	464 ± 13.27 cd	197.28 ± 6.20 bc	177.11 ± 3.16 bc	39.95 ± 1.41 a
	M2T1	17.2 ± 0.48 ab	1.4 ± 0.04 d	48.97 ± 0.10 ab	492 ± 13.05 abc	205.76 ± 6.72 ab	186.33 ± 4.67 ab	38.63 ± 0.84 a
	M3T1	17.7 ± 0.28 a	1.0 ± 0.03 ef	48.01 ± 0.95 ab	518 ± 15.52 ab	212.57 ± 4.17 a	191.59 ± 4.88 a	37.80 ± 1.14 ab
	M4T1	16.2 ± 0.19 bcd	1.6 ± 0.05 c	46.97 ± 0.89 abc	504 ± 17.39 abc	179.23 ± 2.24 de	161.60 ± 5.14 d	34.00 ± 0.99 cd
	M5T1	15.3 ± 0.35 cd	1.9 ± 0.04 b	44.18 ± 1.14 c	418 ± 12.42 e	143.04 ± 3.51 f	130.82 ± 2.02 e	31.85 ± 0.35 de
	M1T2	16.0 ± 0.34 bcd	1.5 ± 0.06 cd	50.48 ± 0.60 a	460 ± 4.01 cde	190.04 ± 0.93 cd	167.82 ± 4.53 cd	37.69 ± 0.72 ab
	M2T2	16.2 ± 0.55 bcd	1.1 ± 0.03 f	49.45 ± 1.02 ab	488 ± 16.31 bc	200.46 ± 6.14 abc	178.92 ± 4.69 abc	37.20 ± 0.88 ab
	M3T2	16.8 ± 0.35 ab	1.2 ± 0.04 e	48.12 ± 1.34 ab	534 ± 13.53 a	208.34 ± 4.76 ab	186.84 ± 4.74 ab	35.56 ± 0.40 bc
	M4T2	16.2 ± 0.44 bcd	1.6 ± 0.06 c	47.52 ± 1.43 abc	492 ± 14.42 abc	174.28 ± 3.05 e	156.73 ± 2.83 d	33.17 ± 0.88 cde
	M5T2	15.0 ± 0.14 d	2.1 ± 0.02 a	46.39 ± 1.30 bc	434 ± 11.59 de	144.24 ± 5.18 f	126.15 ± 1.26 e	30.66 ± 1.05 e
	ANOVA							
	Planting density (M)	9.01 ***	125.09 ***	5.92 **	15.61 ***	65.61 ***	71.91 ***	23.62 ***
	Chemical regulation (T)	5.48 *	0.52 ns	1.26 ns	0.08 ns	1.95 ns	5.98 *	7.50 *
	M × T	0.65 ns	10.90 ***	0.28 ns	0.45 ns	0.23 ns	0.13 ns	0.24 ns
2025	M1T1	17.5 ± 0.52 a	1.5 ± 0.03 e	48.08 ± 0.85 ab	518 ± 5.26 b	201.81 ± 4.22 a	185.95 ± 6.05 ab	36.58 ± 0.86 a
	M2T1	16.6 ± 0.36 abc	1.3 ± 0.02 f	47.78 ± 1.44 ab	532 ± 18.19 ab	206.31 ± 6.14 a	188.45 ± 5.43 a	36.41 ± 1.12 a
	M3T1	17.7 ± 0.57 a	0.9 ± 0.01 g	49.86 ± 1.49 ab	558 ± 12.68 a	209.65 ± 6.62 a	189.79 ± 4.54 a	35.35 ± 0.49 ab
	M4T1	16.4 ± 0.40 abc	1.3 ± 0.03 f	45.90 ± 1.40 bc	476 ± 6.74 cd	175.14 ± 2.73 b	160.70 ± 4.70 acd	33.94 ± 0.66 ab
	M5T1	15.9 ± 0.48 bcd	1.7 ± 0.02 d	43.08 ± 1.25 c	476 ± 4.11 cd	147.50 ± 1.31 c	131.31 ± 3.84 e	28.86 ± 0.49 c
	M1T2	15.3 ± 0.45 cd	2.0 ± 0.05 c	50.26 ± 0.85 a	480 ± 10.61 cd	206.61 ± 7.21 a	170.36 ± 4.48 bc	36.50 ± 1.27 a
	M2T2	16.5 ± 0.32 abc	1.5 ± 0.04 e	48.43 ± 1.40 ab	504 ± 12.28 bc	194.49 ± 2.23 a	174.78 ± 5.74 abc	35.79 ± 0.93 ab
	M3T2	17.7 ± 0.39 a	1.0 ± 0.02 g	48.27 ± 1.06 ab	532 ± 12.83 ab	195.63 ± 6.48 a	179.36 ± 7.06 ab	34.63 ± 0.81 ab
	M4T2	16.9 ± 0.25 ab	2.2 ± 0.06 b	48.13 ± 1.14 ab	480 ± 1.33 cd	170.17 ± 4.05 b	152.28 ± 4.68 d	32.95 ± 0.93 b
	M5T2	14.7 ± 0.51 d	2.6 ± 0.09 a	47.54 ± 1.20 ab	464 ± 4.04 d	141.85 ± 3.76 c	123.29 ± 2.88 e	28.19 ± 0.99 c
	ANOVA							
	Planting density (M)	7.77 ***	220.91 ***	3.43 *	18.07 ***	56.59 ***	45.31 ***	26.67 ***
	Chemical regulation (T)	4.80 *	368.73 ***	4.17 ns	9.73 **	4.21 ns	12.29 **	1.20 ns
	M × T	3.16 *	38.73 ***	1.66 ns	1.29 ns	1.13 ns	0.21 ns	0.07 ns

Note: Different lowercase letters in the same column indicate significant differences among the different treatments (*p* < 0.05). *, ** and *** indicate significant differences among the different treatments at the levels of *p* < 0.05, *p* < 0.01 and *p* < 0.001, respectively. ns means not significant at the level of *p* ≥ 0.05.

## Data Availability

The original contributions presented in the study are included in the article, further inquiries can be directed to the corresponding author.
